# SMART-SLE: serology monitoring and repeat testing in systemic lupus erythematosus—an analysis of anti-double-stranded DNA monitoring

**DOI:** 10.1093/rheumatology/kead231

**Published:** 2023-05-19

**Authors:** Ai Li Yeo, Rangi Kandane-Rathnayake, Rachel Koelmeyer, Vera Golder, Worawit Louthrenoo, Yi-Hsing Chen, Jiacai Cho, Aisha Lateef, Laniyati Hamijoyo, Shue-Fen Luo, Yeong-Jian J Wu, Sandra V Navarra, Leonid Zamora, Zhanguo Li, Yuan An, Sargunan Sockalingam, Yasuhiro Katsumata, Masayoshi Harigai, Yanjie Hao, Zhuoli Zhang, B M D B Basnayake, Madelynn Chan, Jun Kikuchi, Tsutomu Takeuchi, Sang-Cheol Bae, Shereen Oon, Sean O’Neill, Fiona Goldblatt, Kristine (Pek Ling) Ng, Annie Law, Nicola Tugnet, Sunil Kumar, Cherica Tee, Michael Tee, Naoaki Ohkubo, Yoshiya Tanaka, Chak Sing Lau, Mandana Nikpour, Alberta Hoi, Michelle Leech, Eric F Morand, for the Asia Pacific Lupus Collaboration

**Affiliations:** School of Clinical Sciences at Monash Health, Faculty of Medicine, Nursing & Health Sciences, Monash University, Clayton, Victoria, Australia; School of Clinical Sciences at Monash Health, Faculty of Medicine, Nursing & Health Sciences, Monash University, Clayton, Victoria, Australia; School of Clinical Sciences at Monash Health, Faculty of Medicine, Nursing & Health Sciences, Monash University, Clayton, Victoria, Australia; School of Clinical Sciences at Monash Health, Faculty of Medicine, Nursing & Health Sciences, Monash University, Clayton, Victoria, Australia; Department of Medicine, Faculty of Medicine, Chiang Mai University, Chiang Mai, Thailand; Division of Allergy, Immunology and Rheumatology, Taichung Veterans General Hospital, Taichung, Taiwan; Rheumatology Divsion, National University Hospital, Singapore; Rheumatology Divsion, National University Hospital, Singapore; Department of Medicine, University of Padjadjaran, Bandung, Indonesia; Department of Rheumatology, Chang Gung Memorial Hospital, Guishan Township, Taiwan; Department of Rheumatology, Chang Gung Memorial Hospital, Guishan Township, Taiwan; Joint and Bone Center, University of Santo Tomas Hospital, Manila, Philippines; Joint and Bone Center, University of Santo Tomas Hospital, Manila, Philippines; Department of Rheumatology and Immunology, People’s Hospital Peking University Health Sciences Centre, Beijing, China; Department of Rheumatology and Immunology, People’s Hospital Peking University Health Sciences Centre, Beijing, China; Department of Medicine, University of Malaya, Kuala Lumpur, Malaysia; Institute of Rheumatology, Tokyo Women’s Medical University, Tokyo, Japan; Institute of Rheumatology, Tokyo Women’s Medical University, Tokyo, Japan; Rheumatology and Immunology Department, Peking University First Hospital, Beijing, China; Rheumatology and Immunology Department, Peking University First Hospital, Beijing, China; Division of Nephrology, Teaching Hospital, Kandy, Sri Lanka; Department of Rheumatology, Allergy and Immunology Tan Tock Seng Hospital, Singapore; Division of Rheumatology, Department of Internal Medicine, Keio University, Tokyo, Japan; Division of Rheumatology, Department of Internal Medicine, Keio University, Tokyo, Japan; Department of Rheumatology, Hanyang University Hospital for Rheumatic Diseases and Hanyang University Institute for Rheumatology Research and Hanyang University Institute of Bioscience and Biotechnology, Seoul, South Korea; Department of Medicine, The University of Melbourne at St Vincent’s Hospital, Fitzroy, Victoria, Australia; Rheumatology Department, Level 1 Liverpool Hospital, Liverpool, NSW, Australia; Rheumatology Unit, Royal Adelaide Hospital and Flinders Medical Centre, Adelaide, South Australia, Australia; Department of Medicine, Waitemata District Health Board, Auckland, New Zealand; Singapore General Hospital, Singapore; Department of Rheumatology, Auckland District Health Board, Auckland, New Zealand; Department of Rheumatology, Middlemore Hospital, Auckland, New Zealand; University of the Philippines, Quezon City, Philippines; University of the Philippines, Quezon City, Philippines; The First Department of Internal Medicine, University of Occupational and Environmental Health, Kitakyushu, Japan; The First Department of Internal Medicine, University of Occupational and Environmental Health, Kitakyushu, Japan; Division of Rheumatology and Clinical Immunology, University of Hong Kong, Hong Kong, Hong Kong, China; Department of Medicine, The University of Melbourne at St Vincent’s Hospital, Fitzroy, Victoria, Australia; School of Clinical Sciences at Monash Health, Faculty of Medicine, Nursing & Health Sciences, Monash University, Clayton, Victoria, Australia; School of Clinical Sciences at Monash Health, Faculty of Medicine, Nursing & Health Sciences, Monash University, Clayton, Victoria, Australia; School of Clinical Sciences at Monash Health, Faculty of Medicine, Nursing & Health Sciences, Monash University, Clayton, Victoria, Australia

**Keywords:** dsDNA positivity, systemic lupus erythematosus, test monitoring

## Abstract

**Objective:**

Disease activity monitoring in SLE includes serial measurement of anti-double stranded-DNA (dsDNA) antibodies, but in patients who are persistently anti-dsDNA positive, the utility of repeated measurement is unclear. We investigated the usefulness of serial anti-dsDNA testing in predicting flare in SLE patients who are persistently anti-dsDNA positive.

**Methods:**

Data were analysed from patients in a multinational longitudinal cohort with known anti-dsDNA results from 2013 to 2021. Patients were categorized based on their anti-dsDNA results as persistently negative, fluctuating or persistently positive. Cox regression models were used to examine longitudinal associations of anti-dsDNA results with flare.

**Results:**

Data from 37 582 visits of 3484 patients were analysed. Of the patients 1029 (29.5%) had persistently positive anti-dsDNA and 1195 (34.3%) had fluctuating results. Anti-dsDNA expressed as a ratio to the normal cut-off was associated with the risk of subsequent flare, including in the persistently positive cohort (adjusted hazard ratio [HR] 1.56; 95% CI: 1.30, 1.87; *P* < 0.001) and fluctuating cohort (adjusted HR 1.46; 95% CI: 1.28, 1.66), both for a ratio >3. Both increases and decreases in anti-dsDNA more than 2-fold compared with the previous visit were associated with increased risk of flare in the fluctuating cohort (adjusted HR 1.33; 95% CI: 1.08, 1.65; *P* = 0.008) and the persistently positive cohort (adjusted HR 1.36; 95% CI: 1.08, 1.71; *P* = 0.009).

**Conclusion:**

Absolute value and change in anti-dsDNA titres predict flares, including in persistently anti-dsDNA positive patients. This indicates that repeat monitoring of dsDNA has value in routine testing.

Rheumatology key messages

- Anti-dsDNA results can fluctuate, but are persistently positive in nearly one-third of SLE patients.
- Anti-dsDNA results predict flares, including in patients who are persistently positive.
- Larger fluctuations in anti-dsDNA are more predictive of flares.

## Introduction

SLE is a chronic autoimmune multisystem condition with a preponderance for affecting women of childbearing age. Higher disease activity, glucocorticoid exposure and increased numbers of flares have each been demonstrated to increase the risk of irreversible organ damage [1–4]. Therefore, SLE patients require long term monitoring of disease activity in order to reduce the development of damage [5, 6]. Disease activity monitoring has been difficult to standardize given the heterogeneous nature of SLE; some patients have only mild manifestations with no evidence of damage, whereas others have severe disease with end stage organ damage [7, 8]. Monitoring patients using tools that predict flare is potentially of value to identify addressable risk factors for poor outcomes.

Several studies have sought to determine whether biomarkers can predict flare in SLE [9–13], including titres of autoantibodies to double stranded DNA (anti-dsDNA) [12, 14]. However, studies of flare prediction lack standardized methodology, and analyses of cross-sectional data have limited ability to guide strategies in the clinic [15]. Furthermore, some patients are persistently serologically active, and the utility of repeating anti-dsDNA tests in this context is poorly understood, while a subgroup of SLE patients are clinically quiescent despite being serologically active, and fluctuations in anti-dsDNA and complement do not appear to predict flare in these patients [16, 17]. Lastly, providers use different assays to measure anti-dsDNA, thus encumbering standardization, monitoring and cross-referencing [18].

Despite these limitations, anti-dsDNA measurement is often performed repeatedly during SLE management. Widely used disease activity indices such as the SLE disease activity index (SLEDAI) require anti-dsDNA antibody test results to assign a score [19]. Attainment of the lupus low disease activity state (LLDAS) has been demonstrated to reduce damage accrual in SLE [20–23], but SLEDAI is included in the definition of LLDAS, meaning serological testing is required to assess LLDAS attainment. As new treatments for SLE emerge, and treatment targets become part of routine practice, SLEDAI or other formal disease activity measurements may be required as part of reimbursed drug access, meaning the potential for a surge in the serial use of these tests, despite limited understanding about cost-effectiveness. We have recently reported very low utility of repeat testing for other common autoantibodies, anti-nuclear antibodies and extractable nuclear antibodies, albeit in the setting of diagnostic screening rather than monitoring of established disease [24, 25]. Should anti-dsDNA antibodies be serially tested in patients in whom the result is always positive?

In this study, we investigated the utility of serial anti-dsDNA testing in predicting the risk of flare in SLE patients. The focus was on those patients who were persistently anti-dsDNA positive, in order to determine whether the need for serial testing could be obviated in this group.

## Methods

### Study design, setting and participants

Data from the Asia Pacific Lupus Collaboration (APLC) cohort were used to conduct this study [26]. This is a multi-country longitudinal registry of patient data collected prospectively across 25 sites in 13 countries. All patients met either the 1997 ACR Modified Classification Criteria [27] or the 2012 SLICC Classification Criteria [28] for SLE and provided written consent to be part of the APLC cohort. Each site obtained local ethics approval to participate in the APLC research activities. Storage of the central dataset and analyses of the pooled data have been approved by the Monash University Human Research Ethics Committee (MUHREC Project ID 18778).

#### Data collection

Data were collected during routine visits for patient care via electronic point of care data entry or paper or electronic forms from 2013 until 2021. Baseline data collected included age, duration of SLE, sex, ethnicity, baseline serology and comorbidities. At each visit SLEDAI-2K [29], physician global assessment (PGA, measured 0–3) [30], medication information and pathology results including serology were collected. Only visits where an anti-dsDNA result was available were included in analysis, and only patients who had at least two such visits were included. Flares were routinely captured at each visit using the Safety of Estrogens in Lupus Erythematosus National Assessment–SLEDAI flare index (SFI) [30, 31]. This captures flare using a composite of the following: (i) increases of at least 3 points in the SELENA-SLEDAI disease activity instrument, (ii) other new or worsening activity, medication changes and hospitalization not been captured in the SELENA-SLEDAI, and (iii) an increase in the physician’s global assessment (visual analogue scale) ≥1.0. The severity of flare was captured as previously defined by Petri *et al.* [31]. Damage accrual was recorded at baseline and annually using the SLICC-ACR Damage Index (SDI) [32]. Patients were categorized based on their anti-dsDNA results into three cohorts: persistently negative (patients who never had anti-dsDNA values above upper limit of normal range), fluctuating (anti-dsDNA results varying between negative and positive) and persistently positive (patients who always had anti-dsDNA results above upper limit of normal range).

### Standardising anti-dsDNA results

Data on the specific assay performed at each site and across time were not available. Due to the multiple sites and assays used to determine the anti-dsDNA result, we standardized results by calculating an anti-dsDNA ratio, dividing the anti-dsDNA result by the upper limit of normal for that test. This ratio was then used to determine the relative change in anti-dsDNA result between tests, recorded as a vector (value and direction of change) rather than absolute value (Supplementary Fig. S1, available at *Rheumatology* online).

### Statistical analysis

Statistical analysis was performed using Stata Version 15 (StataCorp, College Station, TX, USA).

Continuous variables are described as either mean (standard deviation [SD]) or median (interquartile range [IQR]) depending on data distribution. Medians were compared using the Kruskal–Wallis (comparison of medians) test among anti-dsDNA persistently negative, fluctuating and persistently positive groups. Categorical variables are described as frequency and percentage and compared using the chi-square test.

Cox regression models were used to examine longitudinal associations of anti-dsDNA results and other factors associated with flare (mild-moderate or severe) at the subsequent visit in the persistently positive and fluctuating cohorts. For analysis of change in anti-dsDNA, the anti-dsDNA ratios calculated at two consecutive visits were used to assess prediction of flare assessed at a third consecutive visit (Supplementary Fig. S1, available at *Rheumatology* online). Each univariable Cox regression model contains one dependent (or exposure) variable only. If dependent variables demonstrated univariable associations with *P*-values <0.1, they were included in multivariable regression models. Prentice, Williams and Peterson modelling with gap time (PWP-GT) was incorporated when setting up data to allow multiple ‘failures’, i.e. flare recurrence. In addition, clustering was specified in these Cox regression models to account for intragroup correlation. The results from the time-dependent Cox regression analyses are presented as hazard ratios with corresponding 95% CI. A *P*-value ≤0.05 was considered statistically significant.

## Results

The APLC 2013–2020 dataset included 3811 patients with longitudinal data. Of these, 327 patients did not have anti-dsDNA results, and were therefore excluded from this study, and therefore 3484 patients were studied (Table 1). Of these patients, 3216 (92.3%) were female, with a median (IQR) disease duration at enrolment of 8.0 (3.0, 15.0) years and median (IQR) age at enrolment 39.0 (30.0, 50.0) years; 3092 (89.0%) patients were of Asian ethnicity. Patients had a median (IQR) follow-up of 3 (1, 5) years, with a median of 3.7 (2.6, 4.7) visits per year and a median total number of visits per patient of 9 (5, 15), totalling 37 582 visits.

**Table 1.** Baseline demographic and clinical characteristics of SLE patients categorized as anti-dsDNA persistently negative, fluctuating or persistently positive

		Anti-dsDNA			
	Total (*n* = 3484)	Persistently negative (*n* = 1260)	Fluctuating (*n* = 1195)	Persistently positive (*n* = 1029)	*P*-value^a^
Sex, female, *n* (%)	3216 (92.3)	1161 (92.1)	1105 (92.5)	949 (92.2)	0.97
Age at enrolment, median (IQR), years	39.0 (30.0, 50.0)	44.0 (34.0, 55.0)	37.0 (29.0, 48.0)	37.0 (28.0, 46.0)	<0.001
Disease duration, median (IQR), years	8.0 (3.0, 15.0)	9.0 (3.0, 16.0)	7.0 (2.0, 13.0)	8.0 (3.0, 14.0)	<0.001
Total follow-up duration, median (IQR), years	3.0 (1.1, 5.5)	2.2 (1.0, 5.0)	4.2 (2.3, 6.5)	2.0 (1.0, 4.8)	<0.001
Number of visits per year, median (IQR)	3.7 (2.6, 4.7)	3.8 (2.6, 4.7)	3.6 (2.6, 4.3)	3.9 (2.6, 5.0)	<0.001
Ethnicity, Asian, *n* (%)	3092 (89.0)	1117 (89.1)	1076 (90.1)	899 (87.6)	0.170
ANA positivity, *n* (%)	3286 (94.3)	1170 (92.9)	1119 (93.6)	997 (96.9)	<0.001
ACR criteria, *n* (%)					
Malar rash	1898 (54.5)	646 (51.3)	686 (57.4)	566 (55.0)	0.009
Discoid rash	554 (15.9)	208 (16.5)	203 (17.0)	143 (13.9)	0.110
Oral ulcers	1114 (32.0)	372 (29.5)	404 (33.8)	338 (32.8)	0.058
Photosensitivity	1049 (30.1)	367 (29.1)	372 (31.1)	310 (30.1)	0.56
Arthritis	2284 (65.6)	780 (61.9)	780 (65.3)	724 (70.4)	<0.001
Serositis	623 (17.9)	191 (15.2)	220 (18.4)	212 (20.6)	0.003
Renal	1658 (47.6)	605 (48.0)	595 (49.8)	458 (44.5)	0.042
Neurological	324 (9.3)	130 (10.3)	117 (9.8)	77 (7.5)	0.052
Haematological	2047 (58.7)	718 (57.0)	738 (61.8)	591 (57.4)	0.033
Immunological	3117 (89.4)	1034 (82.1)	1077 (90.1)	1029 (100.0)	<0.001

a For categorical variables, the chi square test was used, for continuous variables, the Kruskal–Wallis test. IQR: interquartile range.

Three patterns of anti-dsDNA results over time were observed, i.e. persistently negative (1260 patients [36%]), fluctuating between negative and positive (1195 [34%]) and persistently positive (1029 [29%]). Demographic and clinical characteristics of these groups are shown in Table 1. Median (IQR) disease duration at enrolment was shorter in fluctuating (7.0 [2.0, 13.0] years) and persistently positive patients (8.0 [3.0, 14.0] years) compared with persistently negative patients (9.0 [3.0, 16.0] years), *P* < 0.001). As captured by baseline ACR criteria, fluctuating and persistently positive cohorts had a higher percentage of patients with arthritis (780 [65.3%] and 724 [70.4%] *vs* 780 [61.9%], *P* < 0.001) and serositis (220 [18.4%] and 212 [20.6%] *vs* 191 [15.2%], *P* = 0.003) than persistently negative patients (Table 2). Persistently positive patients had less baseline renal involvement (458 [44.5%]) per ACR criteria than fluctuating (595 [49.8%]) and persistently negative patients (605 [48.0%]) (*P* = 0.042), while the fluctuating cohort had a higher percentage of patients with haematological manifestations than the other two groups (738 [61.8%] *vs* 718 [57.0%] and 591 [57.4%], *P* = 0.033) (Table 1).

**Table 2.** Medication usage ever in SLE patients categorized as anti-dsDNA persistently negative, fluctuating or persistently positive

		Anti-dsDNA			
	Total (*n* = 3484)	Persistently negative (*n* = 1260)	Fluctuating (*n* = 1195)	Persistently positive (*n* = 1029)	*P*-value^a^
Prednisolone use ever, *n* (%)	2987 (85.7)	987 (78.4)	1081 (90.5)	919 (89.3)	<0.001
Cumulative prednisolone dose, median [IQR] (range), g	4.38 [1.24, 10.47] (0, 106.26)	2.84 [0.36, 7.66] (0, 34.70)	7.19 [2.66, 13.29] (0, 101.19)	4.05 [1.49, 9.96] (0, 106.26)	<0.001
Antimalarials, *n* (%)	2736 (78.5)	915 (72.6)	942 (78.8)	879 (85.4)	<0.001
Methotrexate, *n* (%)	284 (8.2)	85 (6.7)	103 (8.6)	96 (9.4)	0.061
Azathioprine, *n* (%)	1113 (31.9)	289 (22.9)	437 (36.6)	387 (37.6)	<0.001
Mycophenolate, *n* (%)	1296 (37.2)	359 (28.5)	555 (46.4)	382 (37.1)	<0.001
Leflunomide, *n* (%)	90 (2.6)	25 (2.0)	43 (3.6)	22 (2.1)	0.027
Ciclosporin, *n* (%)	293 (8.4)	98 (7.8)	131 (11.0)	64 (6.2)	<0.001
Tacrolimus, *n* (%)	155 (4.4)	58 (4.6)	54 (4.5)	43 (4.2)	0.880
Cyclophosphamide, *n* (%)	307 (8.8)	70 (5.6)	170 (14.2)	67 (6.5)	<0.001
Rituximab, *n* (%)	75 (2.2)	11 (0.9)	32 (2.7)	32 (3.1)	<0.001
Belimumab, *n* (%)	67 (1.9)	13 (1.0)	28 (2.3)	26 (2.5)	0.010

a Medication use was recorded if a medication was used at any time during observation. For categorical variables, the chi square test was used, for continuous variables, the Kruskal–Wallis test. IQR: interquartile range.

The persistently negative cohort had less medication use during the observation period compared with the fluctuating and persistently positive anti-dsDNA cohort (Table 2), including a lower likelihood of prednisolone use (987 [78.4%] *vs* 1081 [90.5%] and 919 [89.3%], respectively *P* < 0.001) and lower median (IQR) cumulative prednisolone dose (g) (2.84 [3.65, 7.66] *vs* 7.19 [2.66, 13.29] and 4.05 [1.49, 10.0], respectively *P* < 0.001). Antimalarial usage was more frequent in the persistently positive cohort compared with fluctuating and persistently negative cohorts (879 [85.4%] *vs* 942 [78.8%] and 915 [72.6%] respectively, *P* < 0.001) (Table 3).

**Table 3.** Disease activity and flare in SLE patients categorized as anti-dsDNA persistently negative, fluctuating or persistently positive

		Anti-dsDNA			
	Total (*n* = 3484)	Persistently negative (*n* = 1260)	Fluctuating (*n* = 1195)	Persistently positive (*n* = 1029)	*P*-value^a^
Time-adjusted mean SLEDAI-2K, median [IQR] (range)	3.0 [1.5, 4.8] (0, 22.0)	1.4 [0.3, 2.8] (0, 13.3)	3.3 [2.0, 5.1] (0, 19.5)	4.3 [3.2, 6.0] (0, 22.0)	<0.001
Time-adjusted mean SLEDAI-2K excluding anti-dsDNA result, median [IQR] (range)	2.0 [0.6, 3.4] (0, 20)	1.1 [0.1, 2.5] (0, 13.3)	2.1 [0.9, 3.7] (0,18.5)	2.3 [1.2, 4.0] (0, 20)	<0.001
Time-adjusted mean PGA, median [IQR] (range)	0.4 [0.2, 0.7] (0, 3.0)	0.3 [0.1, 0.6] (0, 2.4)	0.4 [0.3, 0.8] (0, 2,5)	0.6 [0.3, 0.9] (0, 3.0)	<0.001
Baseline SDI > 1, *n* (%)	1250 (38.4)	493 (41.3)	439 (38.9)	318 (34.2)	0.003
Number of flares during the observation period, median [IQR] (range)	1 [1, 2] (0, 36)	0 [0, 1] (0, 16)	1 [0, 4] (0, 36)	1 [0, 2] (0, 31)	<0.001
0 flares, *n* (%)	1496 (42.9)	685 (54.4)	365 (30.5)	446 (43.3)	<0.001
1 flare, *n* (%)	777 (22.3)	273 (21.7)	236 (19.7)	268 (26.0)	0.001
2 or more flares, *n* (%)	1211 (34.8)	302 (24.0)	594 (49.7)	315 (30.6)	<0.001
Mild/moderate flare, median [IQR] (range)	1 [0, 2] (0, 16)	0 [0, 1] (0, 13)	1 [0, 3] (0, 14)	1 [0, 2] (0, 16)	<0.001
0 flares, *n* (%)	1599 (45.9)	708 (56.2)	397 (33.2)	494 (48.0)	<0.001
1 flare, *n* (%)	820 (23.5)	286 (22.7)	263 (22.0)	271 (26.3)	0.038
2 or more flares, *n* (%)	1065 (30.6)	266 (21.1)	535 (44.8)	264 (25.7)	<0.001
Severe flare, median [IQR] (range)	0 [0, 1] (0, 29)	0 [0, 0] (0, 12)	0 [0, 1] (0, 29)	0 [0, 1] (0, 24)	<0.001
0 flares, *n* (%)	2544 (73.0)	1059 (84.0)	734 (61.4)	751 (73.0)	<0.001
1 flare, *n* (%)	475 (13.6)	108 (8.6)	208 (17.4)	159 (15.5)	<0.001
2 or more flares, *n* (%)	465 (13.3)	93 (7.4)	253 (21.2)	119 (11.6)	<0.001
Annual total flare rate^b^, median [IQR]	0.3 [0.0, 0.9]	0.0 [0.0, 0.6]	0.4 [0.0, 0.9]	0.3 [0.0, 1.0]	<0.001
Annual severe flare rate, median [IQR]	0.0 [0.0, 0.1]	0.0 [0.0, 0.0]	0.0 [0.0, 0.3]	0.0 [0.0, 0.2]	<0.001

a For categorical variables, the chi square test was used, for continuous variables, the Kruskal–Wallis test.

b Flare rate was calculated by dividing the total number of flares by the observation period. IQR: interquartile range; PGA: physician global assessment; SDI: Systemic Lupus International Collaborating Clinics/American College of Rheumatology (SLICC/ACR) Damage Index; SLEDAI-2K: Systemic Lupus Erythematosus Disease Activity Index 2000.

Disease activity also varied between the cohorts. Time-adjusted mean SLEDAI-2K (AMS) was highest in persistently anti-dsDNA positive patients (median [IQR] 4.3 [3.2, 6.0]) and lowest in persistently anti-dsDNA negative patients (median [IQR] 1.4 [0.3, 2.8]) (*P* < 0.001) (Table 3). As anti-dsDNA contributes 2 points to the SLEDAI-2K, we subtracted this domain and found that time-adjusted mean SLEDAI-2K excluding anti-dsDNA remained highest in the persistently anti-dsDNA positive cohort (median [IQR] 2.3 [1.2, 4.0] *vs* persistently anti-dsDNA negative 1.1 [0.1, 2.5], fluctuating 2.1 [0.9, 3.7] and total cohort 2.0 [0.6, 3.4], *P* < 0.001). Persistently anti-dsDNA negative patients were more likely to have damage at baseline compared with fluctuating and persistently positive patients with 493 (41.3%) patients having a baseline SDI ≥1 compared with fluctuating (439 [38.9%]) and persistently positive (318 [34.2%]) (*P* = 0.003) cohorts (Table 3).

A total of 5097 flares were recorded in 1988 (57.1%) patients during the observation period, with a range of 0–36 flares per patient; 1211 (34.8%) of patients experienced recurrent flares; 1255 flares occurred in the persistently anti-dsDNA positive cohort (583/1029 patients [56.7%]) compared with 2613 flares in the fluctuating cohort (830/1195 patients [69.5%]); and 594 (49.7%) fluctuating patients had recurrent flares (≥2) during the observation period, a higher proportion compared with patients who were persistently positive (315 [30.6%]) or persistently negative (302 [24.0%]) (*P* < 0.001) (Table 3).

In univariable Cox regression analyses, disease activity and laboratory values at each visit were analysed to determine whether they predicted flare at the subsequent visit. To determine the association of anti-dsDNA as a continuous variable with risk of flare, two approaches were used: absolute value of anti-dsDNA ratio, and change in anti-dsDNA ratio from the previous visit, recorded as amplitude and direction.

The results of the Cox regression analyses of associations with flare for the whole cohort, including patients who were persistently anti-dsDNA negative, are shown in Supplementary Tables S1 and S2, available at *Rheumatology* online. As the primary aim was to determine whether serial anti-dsDNA monitoring in patients who are persistently anti-dsDNA positive had utility, we focused on these patients. As an absolute value, anti-dsDNA ratio >3 was significantly associated with an increased risk of flare at the subsequent visit in persistently anti-dsDNA positive patients (unadjusted HR [95% CI] 1.64 [1.40, 1.91], *P* < 0.001) (Table 4). In the persistently anti-dsDNA positive cohort, change in anti-dsDNA ratio of at least 2-fold in a positive or negative direction had an unadjusted HR (95% CI) for flare of 1.35 (1.09, 1.68) (*P* = 0.007) and 1.29 (1.05, 1.59) (*P* = 0.014), respectively (Table 4). An increase or decrease of <2-fold did not predict flare.

**Table 4.** Unadjusted hazard ratio of associations with flare^a^

	Fluctuating anti-dsDNA (*n* = 1195)		Persistently anti-dsDNA positive (*n* = 1029)	
	Hazard ratio^b^ (95% CI)	*P*-value	Hazard ratio^b^ (95% CI)	*P*-value
Total number of flares	2613		1255	
Age at enrolment	0.987 (0.98, 0.99)	<0.001	0.98 (0.98, 0.99)	<0.001
Sex				
Female	1		1	
Male	1.06 (0.92, 1.22)	0.405	1.15 (0.95, 1.40)	0.158
Disease duration at enrolment	0.99 (0.99, 1.00)	0.004	0.97 (0.96, 0.97)	<0.001
Baseline SDI score 1 or above	1.11 (1.03, 1.20)	0.007	1.01 (0.96, 1.06)	0.692
SLEDAI-2K activity by category at previous visit				
Central nervous system	1.52 (0.94, 2.45)	0.086	1.54 (0.80, 2.97)	0.199
Renal	1.55 (1.43, 1.69)	<0.001	1.83 (1.62, 2.07)	<0.001
Musculoskeletal	1.23 (1.03, 1.47)	0.024	1.09 (0.87, 1.36)	0.472
Vasculitis	1.61 (1.14, 2.29)	0.007	0.92 (0.46, 1.85)	0.814
Cutaneous	1.28 (1.14, 1.49)	<0.001	1.36 (1.18, 1.58)	<0.001
Serositis	1.93 (1.29, 2.89)	0.001	1.95 (1.19, 3.19)	0.008
Fever	1.39 (0.77, 2.52)	0.273	1.29 (0.58, 2.87)	0.540
Haematological	1.17 (1.00, 1.36)	0.052	0.78 (0.62, 0.97)	0.028
Prednisolone ≥7.5 mg at previous visit	1.61 (1.48, 1.74)	<0.001	1.69 (1.51, 1.89)	<0.001
Anti-dsDNA ratio at previous visit				
≤ 1	1.00			
>1–2	1.06 (0.96, 1.16)	0.246	1.00	
2–3	1.44 (1.27, 1.63)	<0.001	1.35 (1.12, 1.62)	0.001
>3	1.64 (1.46, 1.83)	<0.001	1.64 (1.40, 1.91)	<0.001
Change in anti-dsDNA ratio compared to previous visit^c^				
≤1-fold	1.00		1.00	
Increased 1–2-fold	1.22 (1.00, 1.46)	0.043	1.25 (0.99, 1.58)	0.065
Decreased 1–2-fold	1.08 (0.88, 1.31)	0.471	0.92 (0.72, 1.195)	0.549
Increased >2-fold	1.59 (1.32, 1.92)	<0.001	1.35 (1.09, 1.68)	0.007
Decreased >2-fold	1.41 (1.16, 1.70)	<0.001	1.29 (1.05, 1.59)	0.014
Other laboratory findings				
CRP				
<5	1.00		1.00	
5–10	1.35 (1.11, 1.64)	0.003	1.43 (1.12, 1.84)	0.004
10–20	1.43 (1.09, 1.89)	0.011	2.30 (1.72, 3.10)	<0.001
>20	1.50 (1.08, 2.08)	0.015	2.67 (1.89, 3.77)	<0.001
ESR				
0–15	1.00		1.00	
15–25	1.22 (1.08, 1.39)	0.001	1.39 (1.17, 1.66)	<0.001
>25	1.69 (1.54, 1.87)	<0.001	1.63 (1.40, 1.89)	<0.001
C3				
≥0.8 g/l	1.00		1.00	
<0.8 g/l	1.45 (1.35, 1.57)	<0.001	1.25 (1.11, 1.41)	<0.001
C4				
≥0.16 g/l	1.00		1.00	
<0.16 g/l	1.46 (1.33, 1.59)	<0.001	1.19 (1.04, 1.37)	0.010
UPCR				
≤0.03 g/mmol	1.00		1.00	
>0.03 g/mmol	1.92 (1.76, 2.10)	<0.001	1.75 (1.53, 2.00)	<0.001
Lymphocyte count				
≥1 × 10^9^/l	1.00		1.00	
<1 × 10^9^/l	1.37 (1.26, 1.49)	<0.001	1.48 (1.32, 1.66)	<0.001
Platelet count				
≥150 × 10^9^/l	1.00		1.00	
<150 × 10^9^/l	1.22 (1.08, 1.38)	0.001	1.02 (0.84, 1.23)	0.861

a Flare was captured using the Safety of Estrogens in Lupus Erythematosus National Assessment-SLEDAI flare index.

b Time-dependent Cox proportional hazards ratios.

c Anti-dsDNA ratio calculated by taking the result divided by the upper limit of normal for that assay. UPCR: urine protein/creatinine.

In the persistently positive cohort, univariable analysis showed an increased risk of flares with activity in the following SLEDAI domains: renal (unadjusted HR [95% CI] 1.93 [1.62, 2.07]), cutaneous (1.36 [1.18, 1.58]) and serositis (1.95 [1.19, 3.19]). Haematological activity at the previous visit was negatively associated in univariable analysis with subsequent flare (unadjusted HR [95% CI] 0.78 [0.62, 0.97]). Furthermore, in the persistently positive cohort we found that longer disease duration, older age and prednisolone dose >7.5 mg were associated with increased risk of flare. The following laboratory markers were also associated with an increased risk of flare in the persistently positive cohort: CRP >20 (unadjusted HR [95% CI] 2.67 [1.89, 3.77]), ESR >25 (1.63 [1.40, 1.80]), lymphocyte count <1 × 10^9^/l (1.48 [1.32, 1.66]), protein/creatinine ratio >0.03 g/mmol (1.75 [1.53, 2.00]), low C4 (1.19 [1.04, 1.37]) and low C3 (1.25 [1.11, 1.41]).

In the fluctuating cohort, increased risk of flare at the subsequent visit was associated with activity in all domains of SLEDAI-2K except for fever, central nervous system and haematological (Table 4). Similarly, to the persistently positive cohort, elevated CRP (CRP >25, unadjusted HR [95% CI] 1.50 [1.08, 2.08]), ESR >25 (1.69 [1.54, 1.87]) and abnormal protein/creatinine ratio (1.92 [1.76, 2.10]) were associated with an increased risk of flare. Low C3 and C4 were associated with an increased risk of flare with unadjusted HR (95% CI) of 1.45 (1.35, 1.57) and 1.46 (1.33, 1.59) respectively. A normal lymphocyte count was found to be protective with lymphocyte counts <1 × 10^9^/l having an unadjusted HR (95% CI) of 1.37 (1.26, 1.49).

We next adjusted for covariables that we considered important predictors of flare or that had demonstrated significance in univariable analysis. The final four models included the following parameters measured at the previous visit: lymphocyte count <1 × 10^9^/l, prednisolone dose ≥7.5 mg, ESR, cutaneous, serositis and renal activity as measured on SLEDAI-2K. CRP was excluded from the final models due to collinearity with ESR, and the Pr/Cr ratio was excluded due to collinearity with renal activity. Haematological activity, vasculitis activity, central nervous system activity, and C3 and C4 were dropped from the final model as they became insignificant during the multivariable analysis. After multivariable adjustment, associations with flare were observed in persistently anti-dsDNA positive patients and fluctuating patients for both anti-dsDNA ratio as an absolute value and for fold change in anti-dsDNA between visits (Table 5). An absolute anti-dsDNA ratio >3 demonstrated a hazards ratio (95% CI) of 1.35 (1.17, 1.57) in the fluctuating cohort and 1.51 (1.26, 1.81) in the persistently positive cohort. In persistently anti-dsDNA positive patients, a change in anti-dsDNA ratio of at least 2-fold in a positive or negative direction at the prior visit had an adjusted HR (95% CI) for flare of 1.26 (1.00, 1.57) (*P* = 0.050) and 1.36 (1.07, 1.71) (*P* = 0.010), respectively. The fluctuating cohort had a HR (95% CI) of 1.45 (1.14, 1.84) for a 2-fold increase and a HR (95% CI) of 1.35 (1.07, 1.75) for a 2-fold decrease in the anti-dsDNA ratio.

**Table 5.** Adjusted hazard ratios of associations with flare (multivariable analysis)

	Fluctuating anti-dsDNA (*n* = 924)		Persistently anti-dsDNA positive (*n* = 795)	
Adjusted hazard ratio^a^	Hazard ratio^b^ (95% CI)	*P*-value	Hazard ratio^b^ (95% CI)	*P*-value
Number of flares	1566		786	
Anti-dsDNA ratio at previous visit	Model 1		Model 3	
≤1	1.00			
>1–2	1.00 (0.89, 1.13)	0.981	1.00	
2–3	1.22 (1.04, 1.43)	0.016	1.27 (1.02, 1.57)	0.031
>3	1.35 (1.17, 1.57)	<0.001	1.51 (1.26, 1.81)	<0.001
Change in anti-dsDNA ratio compared to previous visit	Model 2		Model 4	
≤1-fold	1.00		1.00	
Increased 1–2-fold	1.09 (0.83, 1.42)	0.542	1.21 (0.93, 1.57)	0.162
Decreased 1–2-fold	1.13 (0.89, 1.45)	0.317	0.81 (0.59, 1.11)	0.192
Increased >2-fold	1.45 (1.14, 1.84)	0.003	1.26 (1.00, 1.58)	0.050
Decreased >2-fold	1.35 (1.07, 1.71)	0.012	1.36 (1.07, 1.71)	0.010

a Hazard ratio adjusted for ESR, prednisolone use of at least 7.5 mg at previous visit, lymphocyte count, evidence of renal, cutaneous or serositis disease activity at previous visit. Central nervous system activity, vasculitis activity, C4 and C3 were dropped from the final model as they became insignificant during multivariable analysis. CRP and Pr/Cr ratio were excluded in the final model due to collinearity with ESR and renal activity respectively.

b Time-dependent Cox proportional hazard ratios.

One hundred and ninety-eight patients (5.7%), despite meeting SLE classification criteria, did not have a record of a positive ANA test result. Analysis after removal of these patients did not impact on the associations of anti-dsDNA with flare (Supplementary Table S3, available at *Rheumatology* online).

## Discussion

Disease activity monitoring in SLE has long been challenging. This is in part due to the spectrum of disease manifestations that present in SLE and in part due to the challenges of finding biomarkers with which to monitor disease activity. In many centres, anti-dsDNA antibodies are measured repeatedly during SLE management, based on studies suggesting they can correlate with or predict disease flare [9–11, 14]. For example, Ho *et al.* described an increase in dsDNA prior to a flare with a subsequent decrease at the time of flare [33]. Gladman *et al.* described a subset of SLE patients who have persistent serological activity but remain clinically quiescent [17], and we and others have shown that such patients are at higher risk of flare than serologically negative patients [34], but whether absolute levels or fluctuations in anti-dsDNA predict disease flare in patients who are persistently positive is poorly understood. Recent studies, albeit in the setting of screening rather than disease monitoring, have highlighted low utility of serial autoantibody testing [24, 25], and the healthcare cost of ongoing testing in patients whose result does not change between positive and negative, a group comprising two-thirds of our cohort, is significant. In the current study, we examined whether quantification of anti-dsDNA could predict increased risk of flare for SLE patients in patients whose results were persistently positive, as well as in SLE patients in general.

The primary aim of this study was to assess the utility of serial dsDNA monitoring in established SLE patients whose results were persistently positive, to determine whether the need for serial testing could be obviated in this group. Patients who were persistently anti-dsDNA positive had a different disease phenotype compared with those who were persistently anti-dsDNA negative, with a shorter disease duration at enrolment, younger age of diagnosis and more musculoskeletal involvement at baseline, in keeping with previous literature [35, 36]. Patients who were persistently anti-dsDNA positive also had higher disease activity over time, including when anti-dsDNA was subtracted from the SLEDAI-2K measure and when disease activity was measured using a physician global assessment. Use of immunosuppressive medications and cumulative prednisolone exposure was lowest in the persistently anti-dsDNA negative cohort, and of note, the cumulative prednisolone dose, and likelihood of cyclophosphamide and mycophenolate use, was highest in the fluctuating cohort. It is not clear if the associations with fluctuating anti-dsDNA reflect the impact of these drugs on anti-dsDNA or disease characteristics intrinsic to these patients that led to these treatments being used. The fluctuating cohort was followed for a longer period of time, influencing cumulative prednisolone dose. Although biological use was infrequent in this cohort, the increased use of B cell-targeting therapies such as rituximab and belimumab in patients who have fluctuating or persistently positive anti-dsDNA reflects the subset of patients that were included in clinical trials [37, 38].

To our knowledge, this is the first study to specifically address the utility of serial anti-dsDNA testing in a large cohort of patients where results are not varying between negative and positive. Our findings demonstrate utility in serial quantification of anti-dsDNA in patients who were persistently positive, by showing that absolute values and changes in anti-dsDNA were predictive of flare. After multivariable adjustment, patients who had an increase in anti-dsDNA of >2-fold had a 26% increased risk of flare. These results are in keeping with some previous studies that have shown an increase in anti-dsDNA can precede a flare, although this has not been demonstrated in all studies [9, 12, 39–42]. Interestingly, the risk of flare was also increased in patients whose anti-dsDNA reduced >2-fold, by 36%, suggesting either that it is the volatility of anti-dsDNA results between visits that is predictive, or potentially that different organ systems are affected differently in relation to anti-dsDNA but captured similarly in the flare instrument used. This was true for patients in the fluctuating cohort and the persistently positive cohort. Deposition of antibodies into specific organs is potentially another explanation for this phenomenon. One previous study has found that a reduction of anti-dsDNA around the time of increased disease activity occurred in a select few patients prior to the initiation of therapy rather than prior to disease flare [43]. This observation was observed particularly for renal flares with the authors of this study hypothesizing that dsDNA deposition in the kidney was responsible for the reduction in titres.

The adjusted hazards ratios between fluctuating and persistently positive groups were similar for both the absolute anti-dsDNA ratio and the change in anti-dsDNA ratio. This suggests that patients who have ever been anti-dsDNA positive represent a subset of patients distinct from those who have always been negative. More recently there has been work looking into inflammatory and regulatory cytokines and their ability to predict flare [44]. Future studies of the association of anti-dsDNA with these cytokines would be of interest.

We also found that an increase in the absolute value of anti-dsDNA at a given time point was associated with the likelihood of flare at the subsequent visit. Patients who have positive anti-dsDNA at baseline have been shown to be more likely to flare [45, 46]. Our findings suggest a role for both the absolute value of anti-dsDNA and the variability of the result in predicting the risk of future flare.

There are several limitations to this study. First, due to the multisite nature of this cohort, there were multiple assays used to measure anti-dsDNA. To attempt to account for this, we analysed all anti-dsDNA results as a ratio to the upper limit of the normal range for each assay. This may not have captured variations in dynamic range between ELISA-based and other assay formats. Furthermore, availability of anti-dsDNA results may influence the treating physician’s clinical assessment, including the PGA [47], and impact the scoring of flares. This study was performed in the Asia–Pacific region, which includes countries with variations in healthcare systems and access to therapies, and ideally findings would be confirmed in independent cohorts.

In conclusion, this study, performed in a large longitudinally followed multinational cohort, suggests that serial monitoring of anti-dsDNA in patients who are persistently anti-dsDNA positive is not redundant. Rather, absolute values and change between visits are useful in predicting flares. Patients who were persistently anti-dsDNA negative were clinically differentiated from persistently positive and fluctuating anti-dsDNA patients, suggesting a different disease trajectory in which other predictors of flare should be sought. Future research into the predictive value of fluctuations in anti-dsDNA in patients who are serologically active but clinically quiescent would be of value.

## Supplementary Material

kead231_Supplementary_Data

## Data Availability

The patient-level data underlying this article cannot be shared publicly due to the privacy of individuals that participated in the study. The data will be shared on a collaborative basis on request to the corresponding author.

## References

[1] Petri M , Purvey S, Fang H, Magder LS. Predictors of organ damage in systemic lupus erythematosus: the Hopkins Lupus Cohort. Arthritis Rheum 2012;64:4021–8. 22932985 10.1002/art.34672 PMC3510359

[2] Hoi A , Koelmeyer R, Bonin J et al Disease course following High Disease Activity Status revealed patterns in SLE. Arthritis Res Ther 2021;23:191. 34261522 10.1186/s13075-021-02572-1 PMC8278658

[3] Ugarte-Gil MF , Acevedo-Vásquez E, Alarcón GS et al; GLADEL. The number of flares patients experience impacts on damage accrual in systemic lupus erythematosus: data from a multiethnic Latin American cohort. Ann Rheum Dis 2015;74:1019–23. 24525909 10.1136/annrheumdis-2013-204620

[4] Apostolopoulos D , Kandane-Rathnayake R, Louthrenoo W et al Factors associated with damage accrual in patients with systemic lupus erythematosus with no clinical or serological disease activity: a multicentre cohort study. Lancet Rheumatol 2020;2:e24–e30. 38258272 10.1016/S2665-9913(19)30105-5

[5] Györi N , Giannakou I, Chatzidionysiou K et al Disease activity patterns over time in patients with SLE: analysis of the Hopkins Lupus Cohort. Lupus Sci Med 2017;4:e000192. 28243457 10.1136/lupus-2016-000192 PMC5307372

[6] Cervera R , Khamashta MA, Font J et al; European Working Party on Systemic Lupus Erythematosus. Morbidity and mortality in systemic lupus erythematosus during a 10-year period: a comparison of early and late manifestations in a cohort of 1,000 patients. Medicine (Baltimore) 2003;82:299–308. 14530779 10.1097/01.md.0000091181.93122.55

[7] Barr SG , Zonana-Nacach A, Magder LS, Petri M. Patterns of disease activity in systemic lupus erythematosus. Arthritis Rheum 1999;42:2682–8. 10616018 10.1002/1529-0131(199912)42:12<2682::AID-ANR26>3.0.CO;2-6

[8] Fernando MMA , Isenberg DA. How to monitor SLE in routine clinical practice. Ann Rheum Dis 2005;64:524–7. 15769911 10.1136/ard.2003.015248 PMC1755465

[9] Gensous N , Marti A, Barnetche T et al; FHU ACRONIM. Predictive biological markers of systemic lupus erythematosus flares: a systematic literature review. Arthritis Res Ther 2017;19:238. 29065901 10.1186/s13075-017-1442-6 PMC5655881

[10] Andrade SDO , Julio PR, Nunes De Paula Ferreira D, Appenzeller S. Predicting lupus flares: epidemiological and disease related risk factors. Expert Rev Clin Immunol 2021;17:143–53. 33393397 10.1080/1744666X.2020.1865156

[11] Ligtenberg G , Arends S, Stegeman CA, de Leeuw K. Predictors of renal flares and long-term renal outcome in patients with lupus nephritis: results from daily clinical practice. Clin Exp Rheumatol 2022;40:33–8. 33822705 10.55563/clinexprheumatol/c58c39

[12] Pan N , Amigues I, Lyman S et al A surge in anti-dsDNA titer predicts a severe lupus flare within six months. Lupus 2014;23:293–8. 24316605 10.1177/0961203313515763

[13] Thanou A , Jupe E, Purushothaman M, Niewold TB, Munroe ME. Clinical disease activity and flare in SLE: current concepts and novel biomarkers. J Autoimmun 2021;119:102615. 33631651 10.1016/j.jaut.2021.102615 PMC8044029

[14] Linnik MD , Hu JZ, Heilbrunn KR et al; LJP 394 Investigator Consortium. Relationship between anti-double-stranded DNA antibodies and exacerbation of renal disease in patients with systemic lupus erythematosus. Arthritis Rheum 2005;52:1129–37. 15818711 10.1002/art.20980

[15] Esdaile JM , Abrahamowicz M, Joseph L et al Laboratory tests as predictors of disease exacerbations in systemic lupus erythematosus. Why some tests fail. Arthritis Rheum 1996;39:370–8. 8607885 10.1002/art.1780390304

[16] Steiman AJ , GLadman DD, Ibañez D, Urowitz MB. Prolonged serologically active clinically quiescent systemic lupus erythematosus: frequency and outcome. J Rheumatol 2010;37:1822–7. 20595281 10.3899/jrheum.100007

[17] Gladman DD , Hirani N, Ibañez D, Urowitz MB. Clinically active serologically quiescent systemic lupus erythematosus. J Rheumatol 2003;30:1960–2. 12966598

[18] Mummert E , Fritzler MJ, Sjöwall C, Bentow C, Mahler M. The clinical utility of anti-double-stranded DNA antibodies and the challenges of their determination. J Immunol Methods 2018;459:11–9. 29807021 10.1016/j.jim.2018.05.014

[19] Isenberg DA , Manson JJ, Ehrenstein MR, Rahman A. Fifty years of anti-ds DNA antibodies: are we approaching journey's end? Rheumatology 2007;46:1052–6. 17500073 10.1093/rheumatology/kem112

[20] Golder V , Huq M, Franklyn K et al Does expert opinion match the operational definition of the Lupus Low Disease Activity State (LLDAS)? A case-based construct validity study. Semin Arthritis Rheum 2017;46:798–803. 28216192 10.1016/j.semarthrit.2017.01.007

[21] Golder V , Kandane-Rathnayake R, Hoi AY et al; Asia-Pacific Lupus Collaboration. Frequency and predictors of the lupus low disease activity state in a multi-national and multi-ethnic cohort. Arthritis Res Ther 2016;18:260. 27829463 10.1186/s13075-016-1163-2 PMC5103412

[22] Franklyn K , Lau CS, Navarra SV et al; Asia-Pacific Lupus Collaboration. Definition and initial validation of a Lupus Low Disease Activity State (LLDAS). Ann Rheum Dis 2016;75:1615–21. 26458737 10.1136/annrheumdis-2015-207726

[23] Golder V , Kandane-Rathnayake R, Huq M et al Lupus low disease activity state as a treatment endpoint for systemic lupus erythematosus: a prospective validation study. Lancet Rheumatol 2019;1:e95–102. 38229349 10.1016/S2665-9913(19)30037-2

[24] Yeo AL , Le S, Ong J et al Utility of repeated antinuclear antibody tests: a retrospective database study. Lancet Rheumatol 2020;2:e412–7. 38273605 10.1016/S2665-9913(20)30084-9

[25] Yeo AL , Leech M, Ojaimi S, Morand E. Utility of repeat extractable nuclear antigen antibody testing: a retrospective audit. Rheumatology (Oxford) 2023;62:1248–53. 35916723 10.1093/rheumatology/keac437

[26] Kandane-Rathnayake R , Golder V, Louthrenoo W et al Development of the Asia Pacific Lupus Collaboration cohort. Int J Rheum Dis 2019;22:425–33. 30398013 10.1111/1756-185X.13431

[27] Hochberg MC. Updating the American College of Rheumatology revised criteria for the classification of systemic lupus erythematosus. Arthritis Rheum 1997;40:1725. 10.1002/art.1780400928 9324032

[28] Petri M , Orbai AM, Alarcon GS et al Derivation and validation of the Systemic Lupus International Collaborating Clinics classification criteria for systemic lupus erythematosus. Arthritis Rheum 2012;64:2677–86. 22553077 10.1002/art.34473 PMC3409311

[29] Touma Z , Urowitz MB, Gladman DD. SLEDAI-2K for a 30-day window. Lupus 2010;19:49–51. 19910390 10.1177/0961203309346505

[30] Buyon JP , Petri MA, Kim MY et al The effect of combined estrogen and progesterone hormone replacement therapy on disease activity in systemic lupus erythematosus: a randomized trial. Ann Intern Med 2005;142:953–62. 15968009 10.7326/0003-4819-142-12_part_1-200506210-00004

[31] Petri M , Buyon J, Kim M. Classification and definition of major flares in SLE clinical trials. Lupus 1999;8:685–91. 10568907 10.1191/096120399680411281

[32] Stoll T , Seifert B, Isenberg DA. SLICC/ACR damage index is valid, and renal and pulmonary organ scores are predictors of severe outcome in patients with Systemic Lupus Erythematosus. Rheumatology 1996;35:248–54. 10.1093/rheumatology/35.3.248 8620300

[33] Ho A , Magder LS, Barr SG, Petri M. Decreases in anti-double-stranded DNA levels are associated with concurrent flares in patients with systemic lupus erythematosus. Arthritis Rheum 2001;44:2342–9. 11665975 10.1002/1529-0131(200110)44:10<2342::aid-art397>3.0.co;2-8

[34] Golder V , Kandane-Rathnayake R, Huq M et al Evaluation of remission definitions for systemic lupus erythematosus: a prospective cohort study. Lancet Rheumatol 2019;1:e103–10. 38229337 10.1016/S2665-9913(19)30048-7

[35] Sarbu MI , Salman-Monte TC, Rubio Muñoz P et al Differences between clinical and laboratory findings in patients with recent diagnosis of SLE according to the positivity of anti-dsDNA by the *Crithidia luciliae* method. Lupus 2015;24:1198–203. 25716418 10.1177/0961203315573852

[36] Gheita TA , Abaza NM, Hammam N et al Anti-dsDNA titre in female systemic lupus erythematosus patients: relation to disease manifestations, damage and antiphospholipid antibodies. Lupus 2018;27:1081–7. 29460701 10.1177/0961203318760209

[37] Singh JA , Shah NP, Mudano AS. Belimumab for systemic lupus erythematosus. Cochrane Database Syst Rev 2021;(2):Cd010668. 33631841 10.1002/14651858.CD010668.pub2 PMC8095005

[38] Wise LM , Stohl W. Belimumab and rituximab in systemic lupus erythematosus: a tale of two B cell-targeting agents. Front Med 2020;7:303. 10.3389/fmed.2020.00303 PMC7338653 32695790

[39] Swaak AJ , Groenwold J, Aarden LA, Statius Van Eps LW, Feltkamp EW. Prognostic value of anti-dsDNA in SLE. Ann Rheum Dis 1982;41:388–95. 6981385 10.1136/ard.41.4.388 PMC1000956

[40] Floris A , Piga M, Cauli A, Mathieu A. Predictors of flares in Systemic Lupus Erythematosus: preventive therapeutic intervention based on serial anti-dsDNA antibodies assessment. Analysis of a monocentric cohort and literature review. Autoimmun Rev 2016;15:656–63. 26921641 10.1016/j.autrev.2016.02.019

[41] Swaak AJ , Groenwold J, Bronsveld W. Predictive value of complement profiles and anti-dsDNA in systemic lupus erythematosus. Ann Rheum Dis 1986;45:359–66. 3487292 10.1136/ard.45.5.359 PMC1001892

[42] Ter Borg EJ , Horst G, Hummel EJ, Limburg PC, Kallenberg CGM. Measurement of increases in anti-double-stranded DNA antibody levels as a predictor of disease exacerbation in systemic lupus erythematosus. Arthritis Rheum 1990;33:634–43. 2346519 10.1002/art.1780330505

[43] Ho A , Magder LS, Barr SG, Petri M. Decreases in anti-double-stranded DNA levels are associated with concurrent flares in patients with systemic lupus erythematosus. Arthritis Rheum 2001;44:2342–9. 11665975 10.1002/1529-0131(200110)44:10<2342::aid-art397>3.0.co;2-8

[44] Munroe ME , Vista ES, Guthridge JM et al Proinflammatory adaptive cytokine and shed tumor necrosis factor receptor levels are elevated preceding systemic lupus erythematosus disease flare. Arthritis Rheumatol 2014;66:1888–99. 24578190 10.1002/art.38573 PMC4128244

[45] Petri M , Singh S, Tesfasyone H, Malik A. Prevalence of flare and influence of demographic and serologic factors on flare risk in systemic lupus erythematosus: a prospective study. J Rheumatol 2009;36:2476–80. 19833757 10.3899/jrheum.090019

[46] Petri MA , Van Vollenhoven RF, Buyon J et al; BLISS-52 and BLISS-76 Study Groups. Baseline Predictors of Systemic Lupus Erythematosus Flares: data From the Combined Placebo Groups in the Phase III Belimumab Trials. Arthritis Rheum 2013;65:2143–53. 23754628 10.1002/art.37995

[47] Aranow C , Askanase A, Oon S et al Laboratory investigation results influence Physician's Global Assessment (PGA) of disease activity in SLE. Ann Rheum Dis 2020;79:787–92. 32241797 10.1136/annrheumdis-2019-216753

