# Supplementary material for: SMART-SLE: serology monitoring and repeat testing in systemic lupus erythematosus—an analysis of anti-double-stranded DNA monitoring
Source: Rheumatology (Oxford). 2023 May 19;63(2):525–33. doi: 10.1093/rheumatology/kead231 (PMC10836977; doi:10.1093/rheumatology/kead231)
Supplement: kead231_Supplementary_Data [file kead231_supplementary_data.docx]

**Supplementary Figure S1. Standardisation of anti-dsDNA results and assessment of change between visits.**

**A.** Table illustrating two patient examples of serial anti-dsDNA results converted to a ratio to the upper limit of the normal range for the assay used, calculation of fluctuation between visits and measurement of SFI. **B.** Graphical representation of data in (A) for patients A and B. **C.** Graphical representation of measurement of SFI at subsequent visit. E.g. anti-dsDNA ratio between Visit 1 and 2 with SFI assessed at Visit 3.

**A.**

| Patient | Visit | Anti-dsDNA result | Upper limit of normal | Anti-dsDNA ratio | Fluctuation in anti-dsDNA ratio between visits | SFI |
| --- | --- | --- | --- | --- | --- | --- |
| A | 1 | 50 | 7 | 7.14 | - | 0 |
| A | 2 | 16 | 7 | 2.29 | -4.86 | 0 |
| A | 3 | 120 | 24 | 5.00 | 2.71 | 0 |
| A | 4 | 20 | 7 | 2.86 | -2.14 | 1 |
| A | 5 | 40 | 24 | 1.67 | -1.19 | 1 |
| B | 1 | 7 | 14 | 0.50 | - | 0 |
| B | 2 | 38 | 14 | 2.71 | 2.21 | 0 |
| B | 3 | 40 | 14 | 2.86 | 0.14 | 1 |
| B | 4 | 20 | 14 | 1.43 | -1.43 | 0 |
| B | 5 | 5 | 14 | 0.36 | -1.07 | 0 |

***** Safety of Estrogens in Lupus Erythematosus National Assessment–SLEDAI flare index

**B**.

**C**


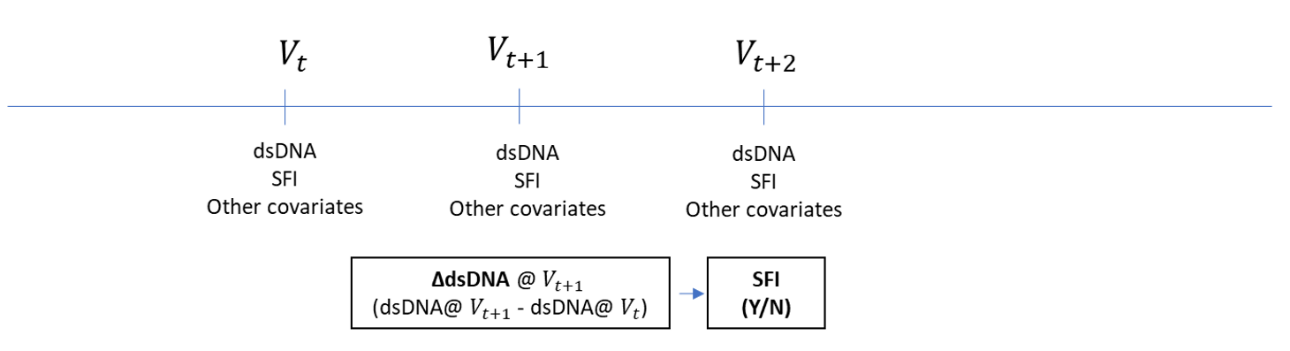


**Supplementary Table S1:** **Unadjusted hazards ratio of associations with flare in entire cohort (n = 3,484)**^§^

| Characteristics | Unadjusted hazard ratio (95% CI) | P value* |
| --- | --- | --- |
|  |  |  |
|  |  |  |
| Age at enrolment | 0.98 (0.98, 0.99) | <0.001 |
| Sex |  |  |
| Female | 1.00 |  |
| Male | 1.08 (0.98, 1.20) | 0.129 |
| Disease duration at enrolment | 0.98 (0.98, 0.98) | <0.001 |
| Baseline SDI score 1 or above | 1.03 (1.01, 1.05) | 0.005 |
| SLEDAI-2K activity by category at previous visit | | |
| Central Nervous System | 1.77 (1.26, 2.48) | 0.001 |
| Renal | 1.75 (1.65, 1.86) | <0.001 |
| Musculoskeletal | 1.33 (1.19, 1.50) | <0.001 |
| Vasculitis | 1.41 (1.05, 1.90) | 0.021 |
| Cutaneous | 1.37 (1.27, 1.48) | <0.001 |
| Serositis | 1.90 (1.42, 2.55) | <0.001 |
| Fever | 1.36 (0.87, 2.14) | 0.179 |
| Haematological | 1.05 (0.93, 1.17) | 0.438 |
| Prednisolone > 7.5mg at previous visit | 1.74 (1.64, 1.84) | <0.001 |
| Anti-dsDNA ratio at previous visit |  |  |
| ≤ 1 | 1.00 |  |
| >1-2 | 1.14 (1.06, 1.23) | 0.001 |
| 2-3 | 1.36 (1.24, 1.49) | <0.001 |
| >3 | 1.49 (1.39, 1.60) | <0.001 |
| Change in anti-dsDNA ratio compared to previous visit^#^ |  |  |
| ≤ 1-fold | 1.00 |  |
| Increased 1-2-fold | 1.34 (1.16, 1.56) | <0.001 |
| Decreased 1-2-fold | 1.11 (0.95, 1.30) | 0.177 |
| Increased >2 fold | 1.58 (1.38, 1.82) | <0.001 |
| Decreased >2 fold | 1.45 (1.27, 1.66) | <0.001 |
| Other laboratory results at previous visit | | |
| CRP |  |  |
| <5 | 1.00 |  |
| 5-10 | 1.43 (1.25, 1.64) | <0.001 |
| 10-20 | 1.88 (1.57, 2.24) | <0.001 |
| >20 | 1.88 (1.52, 2.33) | <0.001 |
| ESR |  |  |
| 0-15 | 1.00 |  |
| 15-25 | 1.22 (1.12, 1.33) | <0.001 |
| >25 | 1.66 (1.54, 1.78) | <0.001 |
| C3 |  |  |
| ≥0.8g/L | 1.00 |  |
| <0.8g/L | 1.42 (1.35, 1.50) | <0.001 |
| C4 |  |  |
| ≥0.16g/L | 1.00 |  |
| <0.16g/L | 1.39 (1.31, 1.48) | <0.001 |
| UPCR |  |  |
| ≤0.03g/mmol | 1.00 |  |
| > 0.03g/mmol | 2.00 (1.87, 2.13) | <0.001 |
| Lymphocyte count |  |  |
| ≥1 x 10^9^/L | 1.00 |  |
| <1 x 10^9^/L | 1.42 (1.34, 1.51) | <0.001 |
| Platelet count |  |  |
| ≥ 150 x 10^9^/L | 1.00 |  |
| <150 x 10^9^/L | 1.16 (1.06, 1.27) | <0.001 |

^^^ Anti-dsDNA = anti-double-stranded DNA. CRP = C-reactive protein. ESR = erythrocyte sedimentation rate. C3 = complement 3. C4 = complement 4. UPCR = urine protein/creatinine

^#^anti-dsDNA ratio calculated by taking the result divided by the upper limit of normal for that assay.

*Time dependent cox proportional hazards ratios

^§^Flare was captured using the Safety of Estrogens in Lupus Erythematosus National Assessment-SLEDAI flare index

**Supplementary Table S2: Adjusted hazards ratio of associations with flare in entire cohort (n=3126)**

| Characteristic | Hazards ratio^¶^ (95% CI) | P value |
| --- | --- | --- |
| Anti-dsDNA ratio at previous visit | ***Model 1*** |  |
| ≤ 1 | 1.00 |  |
| >1-2 | 1.03 (0.94, 1.14) | 0.496 |
| 2-3 | 1.16 (1.03, 1.30) | 0.014 |
| >3 | 1.22 (1.11, 1.34) | <0.001 |
| Change in anti-dsDNA ratio compared to previous visit | ***Model 2*** |  |
| ≤ 1-fold | 1.00 |  |
| Increased 1-2-fold | 1.12 (0.92, 1.38) | 0.256 |
| Decreased 1-2-fold | 1.00 (0.82, 1.21) | 0.973 |
| Increased >2 fold | 1.32 (1.11, 1.58) | 0.002 |
| Decreased >2 fold | 1.31 (1.11, 1.55) | 0.001 |

^¶^ Adjusted for ESR, Prednisolone use of at least 7.5mg at previous visit, lymphocyte count, evidence of renal, cutaneous or serositis disease activity at previous visit. Central nervous system activity, vasculitis activity, complement 4 and complement 3 were dropped from the final model as they had become insignificant during multivariable analysis. CRP and Pr/Cr ratio were excluded in the final model due to collinearity with ESR and renal activity respectively.

**Supplementary Table S3: Associations with flare after removal of ANA-negative patients (multivariable analysis)**

|  | Hazard ratio^#^ (95% CI) | P value | Hazard ratio^#^ (95% CI) | P value |
| --- | --- | --- | --- | --- |
|  | Fluctuating anti-dsDNA (n=871) |  | Persistently anti-dsDNA positive (n=774) |  |
| Number of flares | 1471 |  | 770 |  |
| Anti-dsDNA ratio at previous visit | ***Model 1*** |  | ***Model 3*** |  |
| ≤ 1 | 1.00 |  |  |  |
| >1-2 | 1.01 (0.89, 1.14) | 0.918 | 1.00 |  |
| 2-3 | 1.21 (1.02, 1.43) | 0.024 | 1.30 (1.03, 1.65) | 0.029 |
| >3 | 1.38 (1.19, 1.60) | <0.001 | 1.55 (1.23, 1.97) | <0.001 |
| Change in anti-dsDNA ratio compared to previous visit | ***Model 2*** |  | ***Model 4*** |  |
| ≤ 1-fold | 1.00 |  | 1.00 |  |
| Increased 1-2-fold | 1.03 (0.78, 1.37) | 0.820 | 1.19 (0.92, 1.55) | 0.193 |
| Decreased 1-2-fold | 1.18 (0.92, 1.52) | 0.195 | 0.80 (0.58, 1.10) | 0.178 |
| Increased >2 fold | 1.45 (1.13, 1.85) | 0.003 | 1.23 (1.00, 1.55) | 0.075 |
| Decreased >2 fold | 1.36 (1.07, 1.73) | 0.012 | 1.35 (1.07, 1.71) | 0.013 |

^#^ Hazards ratio adjusted for ESR, Prednisolone use of at least 7.5mg at previous visit, lymphocyte count, evidence of renal, cutaneous or serositis disease activity at previous visit. Central nervous system activity, vasculitis activity, complement 4 and complement 3 were dropped from the final model as they had become insignificant during multivariable analysis. CRP and Pr/Cr ratio were excluded in the final model due to collinearity with ESR and renal activity respectively.
